# Off the Beaten Path: An Interview with Spencer Wells

**DOI:** 10.1371/journal.pgen.0030044

**Published:** 2007-03-30

**Authors:** Jane Gitschier

Spencer Wells has this geneticist's dream job. He transits the globe, collecting DNA samples, building collaborations, and orchestrating what may be the most extensive and fascinating project on human origins yet, sponsored by one of the most respected institutions in the US—The National Geographic Society. There, Wells holds the oxymoronically named post of “Explorer-in-Residence” and runs the Genographic Project (http://www.nationalgeographic.com/genographic), whose mission is to collect and genotype the Y and mitochondrial chromosomes from people the world over in order to track male and female lineages, respectively, and thereby infer migratory patterns throughout human history.

I was curious to learn how someone so young (he started working with National Geographic when he was only 33) could plunge into a project of this magnitude. The answer is that Wells is a man of many facets, vision, and energy. And he has a knack for creating the opportunity.

Happily, I had less difficulty pinning him down than I had anticipated. He suggested getting together at the American Society of Human Genetics meeting in New Orleans, where he was scheduled to give a talk. We held our interview at the open-air Café du Monde in the old French Quarter, where we were embraced by a warm breeze and the cacophony of traffic, tourists, and two busking saxophonists. Our conversation ranged over two cups of café au lait; six continents; and a medley of “The Pink Panther,” “As Time Goes By,” and two rounds of “Somewhere Over the Rainbow.”

Picture, if you will, an Indiana Jones type, passionately delving into ancient mysteries, but in Wells's case, sunburned, hatless, and minus the whip.

**Jane Gitschier:** Let's talk about your transition from academia to adventure.

**Spencer Wells:** Well, it was kind of roundabout. I had done my PhD at Harvard, and like many geneticists in the late '80s and early '90s, I was working on a model organism, and in this case, because my advisor was [Richard] Lewontin, it was *Drosophila*.

I did basic molecular evolution stuff, trying to detect selection in protein coding regions for an enzyme that was important for flight. I looked at variation across species and within species and did detailed statistical analysis. I found no evidence of selection, but lots of evidence of population structure. And at the end of the day I wasn't terribly interested in the population structure of fruit flies, but I had always been interested in human history.

So I wanted to apply those methods to humans, and the technology in genetics was getting to the point where you could start to study human population genetics, because back in the '80s it had been quite difficult. The human genome was so big and you had to clone everything. With PCR it got a lot easier.

Lewontin said, “You've got to work with Luca Cavalli-Sforza,” out at Stanford. When I got to Stanford, one of the first things that Luca said to me was that I needed to get out into the field and meet some of the people that I was thinking of studying. Not only do we need the samples, but it's also important to hear their stories and to get to know them—to become an anthropologist, in effect.

**JG:** Did Luca encourage everyone to do that?

**SW:** He did. I had always been fascinated by Central Asia and I put together my first short expedition there for the summer of 1996. I spent five weeks in Uzbekistan, Kyrgyzstan, and Kazakhstan collecting samples.

**JG:** Wait a second. Just hopping on a plane and going to Uzbekistan in 1996—how did you make those connections?

**SW:** Good question. We knew next to nothing about Central Asia, which is why it was so fascinating. I sent off letters to the US Embassies in all of the “stans”—the newly independent stans—Kazakhstan, Turkmenistan, Tajikistan, Uzbekistan, and Kyrgyzstan. I asked the US Embassies if they could suggest any local scientists who might be interested in working with me on collecting local samples and doing a DNA study.

Most of the embassies came back and said, “No, we can't help, we don't know anybody who would be interested in this.”

But the University of Tashkent, the capital of Uzbekistan, relayed it to the Academy of Sciences. And eventually, Ruslan Ruzibakiev, the director of the Institute of Immunology, happened upon it. He thought it sounded fascinating, so he wrote back to me. That was the start of a great relationship.

**JG:** What exactly did you suggest to him?

**SW:** I told him that we know very little about this very important region of the world. If you look at Eurasia, and you think about people coming out of Africa and populating the planet, this region must have played a very important role—certainly the populating of Asia, but potentially the populating of the Americas as well, and possibly India. But the very little we did know about it suggested that it was unusual. It wasn't just a mix of East and West. There might be a unique indigenous group of people who always lived there.

I told him about the new DNA markers, microsatellites, and SNPs, and this thing called the Y chromosome we were working on at Stanford and how it's starting to reveal some interesting patterns. What if we collected samples and did some studies to try to figure out how these people fit into the world pattern? And he said, “Yeah, sounds amazing. I can set up all the details locally. You deal with all the other logistical stuff, getting yourself over here and funding it, but I know the people we need to contact.”

**JG:** How much later after you had corresponded did you take off?

**SW:** He got back to me early in 1996, and by the summer, after a stack of faxes, I went.

**JG:** How did you fund it?

**SW:** That was funded with money from my Sloan Foundation post-doctoral fellowship. They give you $8,000 for reagents and supplies, and I used about $5,000 to get over there and bring all the equipment.

**JG:** You went by yourself?

**SW:** I went with a photographer, Mark Read, an English friend of my girlfriend at the time. He said, “Nobody has pictures of this part of the world. I'd love to come with you if you wouldn't mind—I have nothing to do this summer.”

So I said, “Yeah, sure, sounds like fun.” So the two of us set off to Central Asia knowing very little about it, speaking very little Russian.

**JG:** Is Russian the language of all those countries?

**SW:** It's the lingua franca because all those countries were part of the Soviet Union.

When we got off the plane, Ruslan picked us up with other members of the Institute. We went to his office—it was 8 o'clock in the morning—he gave us shots of vodka and said, “We have two variants: first variant is we rest today and work tomorrow, and the second variant is work today. I think we'll take the second variant.”

So I was making buffers in the lab and getting ready for the first part of our expedition out of the capital two hours later.

**JG:** Who drew the blood? This always seems like a daunting logistical problem to me.

**SW:** I drew some of the blood, but we mostly worked with local phlebotomists, nurses, and so on. I was taught by one of the nurses there.

**JG:** Did you have consent forms for all these people?

**SW:** We did. We mostly took oral consent but we did have people sign in most cases. We tried very hard to explain what the project was all about.

**JG:** This is a good point for me to ask you about something called your “blood speech.”

**SW:** That's a term we used in *Journey of Man* when we were filming it. [*Journey of Man* is also the title of Wells's expertly written book on the use of DNA to track human origins. His new book on the Genographic Project is called *Deep Ancestry*.]

Most people are interested in their history, and indigenous people, who are the ones who give us the clearest glimpse of their genetic history, are particularly interested, because in many cases it is all they have—what they cling on to—their sense of identity.

I was just in Tajikistan a week and a half ago, and we were sampling all over the southern part of the country and asking people to name their grandparents and great-grandparents and so on. I could do that back to maybe to my great-grandparents. These people can do it back six, seven, eight generations. They've always lived in the same place and beyond that they know even more about their history, but not necessarily their names.

So they have a sense, a clear idea of where they came from, that something is passed from generation that ties them to their ancestors. You explain to them that that thing is DNA and that it will tell us not only about the people they can name but also people beyond that that they can't name, and also people on the other side of the world—me, people you've never met in Africa or southeast Asia, and so on.

People tend to get really excited about that, I find. Generally we get a very positive response. They want to know more. They say, “I'll give you the sample, but make sure you get the information back to me, and tell me what it's all about.”

**JG:** Do you get back to them?

**SW:** Yes, but not necessarily every single person individually. Sometimes we do a press interview and it will be broadcast into an area that we've worked in, and we'll talk about the group and what their DNA samples have told us.

**JG:** So you came back to Stanford with these bloods. Had you extracted DNA as you were collecting?

**SW:** We made a white cell lysate into a high SDS, high Tris buffer, so they were stable in field conditions and didn't have to be refrigerated for weeks or even months.

**JG:** How did you get them all through customs?

**SW:** It turns out there are no regulations about the import of DNA in the United States. DNA samples can be received from anywhere. They can't be sent *out* of China or out of India, there are very strict controls, but in terms of importing DNA, if it's noninfectious, absolutely.

**JG:** Now we're back at Stanford sometime into 1997.

**SW:** And it's clear that we're seeing some interesting stuff in the samples, but we can't make a lot of sense of it because we've got this one little dot on the [geographic] map with a huge area around it that hasn't been sampled. So what we needed to do was increase the sample size from the surrounding regions.

**JG:** And this informed your thinking in going back to Central Asia in 1998. Tell me about it.

**SW:** That we should do it as a huge trip. And we should do it with a media component.

**JG:** Why did you feel that way?

**SW:** Because it's interesting! It's fun to tell people about these things. The Internet was just becoming big then. The BBC had just launched their new Web site, which was quite big in the UK, where Mark Read was based. He knew somebody there who was interested in covering the trip. It was also in part to help attract the funding for it, because a six-month overland expedition is expensive, and you need a vehicle.

So we approached Land Rover, and they were very interested in this and gave us a brand new Discovery. Virgin Atlantic agreed to fly all the equipment and us over to the UK, where we were going to start the trip, for free.

**JG:** Just for people like me who might want to try this kind of approach, how did you pull this off?

**SW:** You write a letter.

**JG:** To whom?

**SW:** To a contact. For Land Rover, it was through a family friend, who knew someone on the board. The letter gets into the right hands. In the case of Virgin Atlantic, it was just writing out of the blue to their marketing or public relations department. Just selling people on a sexy idea. The Web site, I think, did play a role in getting the funding. It provides a place for the person who gave you the equipment to be seen. And it was a new idea at the time. Not a lot of people were doing live expeditions on the Internet.

**JG:** Does Virgin Atlantic fly to the stans?

**SW:** No, we started in London and drove.

**JG:** Oh, God. Why did you do that?

**SW:** Because I liked the challenge of it.

**JG:** Yeah, but how many thousands of extra miles is it?

**SW:** It was also because I liked the idea of seeing the transition from Europe to Asia, and because we were going to start sampling in the Caucuses and the flights would have been very expensive, trying to hop between all these countries and hiring local cars and all of that. It made more sense to have a car once we got there, and it was just a question of getting it there. And it turns out it's a pretty quick drive. If you went straight through it would take you five days to get to the Georgian border.

**JG:** OK, you are planning this trip.

**SW:** I decided to leave Luca's lab to do this trip. It's hard to convince anybody, even Luca, to pay you for six months while you're out in the field. And I didn't know where I was going to end up afterwards.

**JG:** So who actually funded it?

**SW:** Luca's NIH grant did pay for the Vacutainers and the reagents.

I had applied for grants from various organizations and the anthropology funding groups would say, “You're doing genetics, you should be able to get NIH funding for this,” and the NIH comes back and says, “We don't want to do human diversity or human origins research.” And that's part of the reason we're funding Genographic the way we're funding it [partially through public involvement, below].

**JG:** I know the frustration. Who else was on the trip?

**SW:** Nat Pearson, who had been an undergraduate at Stanford and was starting grad school at the University of Chicago that fall. Mark Read. The journalist Darius Bazeran, who left us in Iran, and then Ruslan Ruzibakiev met us in Georgia.

**JG:** Was the journalist posting the things on the Web?

**SW:** He posted stuff for the BBC. The day we left, the BBC had a big story on the front page about “Groundbreaking US/Uzbek team leaves for Central Asia.”

We did the posts [http://popgen.well.ox.ac.uk/eurasia] ourselves—I would write one, then I would edit everything, do the html, upload it, often at four in the morning before we had to leave at six a.m. Then I slept on the road. It was really fun. We had a digital camera and we posted sounds with RealAudio from people singing in cathedrals in Georgia, counting to ten in Lezgi—whatever.

**JG:** So you went around Central Asia, giving the blood speech, collecting blood, engaging people. Six months is a long time!

**SW:** Toward the end, we had two graduate students from Oxford—Matt Webster and Tatiana Zerjal—and they spent two to three weeks with us out in the field to experience it collecting samples themselves. I think they had a good time and learned a lot. That forms the core of what Tatiana did for her thesis on Genghis Khan [i.e., evidence for a common Mongol Y chromosome spread throughout Asia].

**JG:** Was there any point during this at which you felt despair? “Oh my god why am I doing this?”

**SW:** Absolutely. A lot of fieldwork is incredibly boring. A lot of time is spent waiting for permissions, sitting in meetings, waiting for people to show up. Or you blow into town on a Saturday afternoon in one of the ugliest places in the world and you have a day and half to waste.

**JG:** So what did you do?

**SW:** You read or talked. But I did question what the hell I was doing out here. I didn't have a job lined up for when I returned.

Later, a possibility presented itself to work with Walter Bodmer at Oxford. So the samples moved to Walter's lab. And post-docs started coming through.

Then things started to get really exciting scientifically. We had a huge number of samples. In addition to the ones we collected on the trips, our collaborators continued to collect, so we ended up with over 2,000 samples and amazing Y chromosome markers that Peter Underhill and Peter Oefner discovered. The combination was incredible. Every experiment we ran was exciting and new. You're getting these results and they start to make sense. Piecing together migratory patterns. As we started to publish papers, the popular press picks up on it because the Y chromosome is such a great tool for telling these stories and because it actually reveals migratory routes and ties in with historical figures, in the case of Genghis Khan and so on.

Eventually I was contacted by a film production company in London that was working on a film about human variation. Are we all the same, or are we different—the whole race issue. They interviewed me and the producer said, “This stuff is absolutely amazing,” and suggested, “We should do a film just on what you guys are doing,” and that became the *Journey of Man*.

Then, people at National Geographic wanted to have a meeting with me because *Journey of Man* was going to be one of their big television specials. That was the genesis of the Genographic Project. We developed it over the course of the next couple of years, organized the funding, and it is now launched.

**JG:** For our readership—the goal of the Genographic Project is to . . .

**SW:** To use DNA as a tool to answer that basic human question**—**where do we all come from.

**JG:** And the way you are going to do that is . . .

**SW:** Is by studying genetic markers from people from around the world, focusing particularly on indigenous groups because they retain that geographic context in which the genetic patterns originated to a greater extent than people like me—I've got ancestors from all over northern Europe and I live on the eastern seaboard of north America.

**JG:** And you're doing this via collaborations with people all over the world.

**SW:** We have ten regional centers focusing on sampling extant populations. One center is devoted to ancient DNA, which is a very important component of it.

**JG:** Does National Geographic support these people directly?

**SW:** Yes, we essentially give them multi-year grants. They are contracts, and there are deliverables.

**JG:** So these centers do the fieldwork, make the DNA, and do the genotyping, and then?

**SW:** The data are sent to a central database created by IBM. They supplied the server, which is sitting in the basement of National Geographic. They have given everybody laptops with biometric [i.e., fingerprint] recognition so that only the PIs can access to the database. We are working closely with their computational biology team on analyzing the data. So some of our first publications, which are starting to go into the journals now, are coming through that group.

**JG:** Are you at liberty to say what these are about?

**SW:** We are expanding the survey of whole mitochondria genomes in Africa. We've doubled the size of that database and it's revealing interesting mitochondrial patterns. That effort has been spearheaded by Doron Behar in Haifa.

People tend to ignore what went on *within* Africa. There is this inherent bias in European and Asian scientists that we've “done” Africa and *then* things got interesting when we [humans] left, but of course there was still a lot going on within Africa. We're looking at routes people might have taken out of Africa and back migration into Africa. Information that is coming out, in part, from an expedition I organized in 2005 to the Tibesti mountains in Chad, up on the Libyan border.

**JG:** There is also the public participation component of the project [https://www3.nationalgeographic.com/genographic/participate.html]. How many people have sent in their 100 bucks and their cheek swab [for DNA analysis]?

**SW:** Around 165,000. It's been a great response.

**JG:** What is the reaction from people when they get their ancestral information?

**SW:** It runs the gamut from people who write to tell us how amazing the project is and how they've learned what it is to be human to people who say “I already knew I was Western European, you didn't tell me anything new.” But mostly people are very positive about it. I think it is really tapping into something, certainly in North America—this desire to learn more about where we came from. We are a nation of immigrants, so it's not too surprising.

We've gotten some fascinating results and a lot of e-mails. For example, a Hungarian woman wrote in and said, “You've got to redo my test. You told me I'm native American or Siberian, and I know my ancestors came from Hungary—I can tell you the village they were living in in the sixteenth century.” The Hungarian language, Magyar, is actually related to languages spoken in Siberia, and this is one of the first cases where we've actually seen Siberian lineages showing up in the Hungarian population. They are there at very low frequency. We now through this project have over 350 people who are of Hungarian descent and we see these [Siberian] lineages at four to five percent on both male and female sides.

**JG:** What kind of information do people give you when they sign on to this project?

**SW:** It is totally anonymous. We don't actually know which number goes into which kit. You get a randomly generated alpha-numeric code and that is the only way you can access your results on the Web site. When you log on, the first thing that pops up is a sign that says, “please help us with the project by telling us more about yourself and donating your information to the database.” So we ask people their sex, their birth date, zip code, language of their parents, origin of their earliest known male ancestor, and so on. So it provides a little bit of anthropological context.

**JG:** I'd like to do it myself.

**SW:** Well, we can send you a kit.

Public participation has raised around sixteen million gross. The net proceeds are around four million. This is a nonprofit endeavor, and we're plowing that money back into the project, with half going to the field research and the other half going to the “legacy” fund to fund projects by indigenous groups. For example, a project on language preservation, or to fund a Middle Eastern women's cooperative to resurrect a particular type of traditional embroidery work. We're giving our first grants before the end of the year. They are typically around $25,000 each. It's a way to give something back to the indigenous people whose way of life is endangered in some places.

**JG:** Shifting gears, I'm interested in the general question of how people end up doing what they're doing. As a child, what was your thinking?

**SW:** I wanted to be a historian or a writer when I was very young.

**JG:** Why?

**SW:** I was fascinated by history. It was just this idea that there were people who lived in a different way in a different time. And you could imagine yourself, through reading a book, being there with them—like time travel.

I wasn't that interested in science as a kid. I collected rocks and insects just like any other kid, but in terms of a career, I wasn't that excited till my mother went back to graduate school to get her Ph.D. in Biology and I started hanging out in the lab with her and discovered that science was really fun and cool. It's not just about geeky guys in white lab coats, it's about solving puzzles on a daily basis.

I decided I wanted to combine the two in some way. I studied molecular biology in college, at the University of Texas at Austin.

My father is a lawyer, a tax attorney. I was never tempted to follow his footsteps. But he comes from a military family. His father, whom he never knew because he died in World War II, graduated top of his class from West Point and was apparently a wild man out in the field. I think maybe I got some of my love of danger and going to strange places from him.

**Figure:**
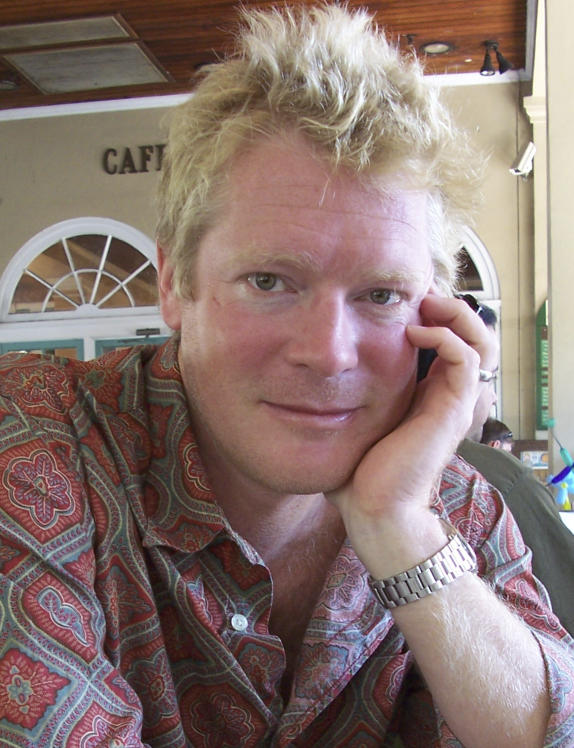
Spencer Wells

**JG:** Give me an example or two.

**SW:** Everything is interesting in its own way. Spending time with the Chukchi reindeer herders, where one morning in Siberia it got down to −70 ^°^C. I had never experienced cold like that—dry ice levels almost. It takes you out of yourself. You think that life is all about certain things, but something like that causes you to sit up and think there are some pretty extreme, amazing things in the world. And the fact that we were there filming people who were living in a traditional way in animal skins and we were in thick down coats—that *really* gives you pause! People have lived like this for tens of thousands of years.

I'm very, very lucky that I have the opportunities that I do, to travel around the world and meet people and hear their stories and see their way of life. It makes me very sad to see that those ways of life are dying.

**JG:** The Genographic Project is a model for doing things outside of a university setting, for getting the public actively involved in a real-time scientific enterprise, and to help fund it. I think it's fantastic.

**SW:** The only advice I would give to young scientists is to think outside the box. Don't just do what your advisor did. Don't just do what the other graduate students are doing. If you have interests that seem a little bit flaky, off the beaten path, that's probably a good thing—especially if they are passions. 

